# 

**Published:** 1900-05

**Authors:** 


*Fifty-Fifth Year.*

VOL. LV.

WHOLE NUMBER,

DCXLII

MAY, 1900.

New Series.

VOL. XXXIX.

No. 10.

### BUFFALO MEDICAL JOURNAL

**Established 1845 by AUSTIN FLINT, M. D.**

**EDITOR:**

WILLIAM WARREN POTTER, M. D.

**ASSISTANT EDITORS:**

WILLIAM C. KRAUSS, M. D. NELSON W. WILSON, M. D.

**ASSOCIATE EDITORS:**

JAMES WRIGHT PUTNAM, M. D. ERNEST WENDE, M. D.
JOHN PARMENTER, M. D. ALVIN A. HUBBELL, M. D.
JOHN A. MILLER, PH. D. MAUD J. FRYE, M. D.

LIBRARY
SURGEON GENERAL'S OFFICE
MAY.-11.-1900

EUROPEAN AGENCY, J. B. BAILLIERE & FILS, 19 Rue Hautefeuille, Paris.
*Subscription, if paid in advance, $2.00; otherwise, $2.50. Foreign subscription, $2.50*
Copyright 1900, by WILLIAM WARREN POTTER, M. D.
Entered at the Post Office at Buffalo, N. Y., as second-class mail matter.

A. T. BROWN PRINTING HOUSE, BUFFALO, N. Y.

**Table of Contents on Page V.**

